# Supplementary material for: Asynchronous magnetic resonance elastography: Shear wave speed reconstruction using noise correlation of incoherent waves
Source: Magn Reson Med. 2022 Oct 27;89(3):990–1001. doi: 10.1002/mrm.29502 (PMC9792433; doi:10.1002/mrm.29502)
Supplement: Supplementary file 1 — DATA S1 MATLAB code used to generate the numerical simulations of Figure 1 [file MRM-89-990-s002.zip › k-Wave/helpfiles/kspaceSecondOrder.html]

kspaceSecondOrder :: Functions (k-Wave)


# kspaceSecondOrder

Fast time-domain simulation of wave propagation for homogeneous media.

## Syntax

```
sensor_data = kspaceSecondOrder(kgrid, medium, source, sensor)
sensor_data = kspaceSecondOrder(kgrid, medium, source, sensor, ...) 

[sensor_data, field_data] = kspaceSecondOrder(kgrid, medium, source, sensor)
[sensor_data, field_data] = kspaceSecondOrder(kgrid, medium, source, sensor, ...)
```

## Description

`kspaceSecondOrder` simulates the time-domain propagation of linear compressional waves through a one, two, or three dimensional homogeneous acoustic medium given four input structures: `kgrid`, `medium`, `source`, and `sensor`. The computation is based on an exact second-order k-space model for media with power law absorption. At each time-step (defined by `kgrid.dt` and `kgrid.Nt` or `kgrid.t_array`), the pressure at the positions defined by `sensor.mask` are recorded and stored. If `kgrid.t_array` is set to `'auto'`, this array is automatically generated using the `makeTime` method of the `kWaveGrid` class. To prevent wave wrapping, the computational domain can be automatically expanded by a factor of two by setting the optional input `'ExpandGrid'` to `true`.

An initial pressure distribution can be specified by assigning a matrix (the same size as the computational grid) of arbitrary numeric values to `source.p0`. An initial pressure gradient can similarly be specified using `source.dp0dt`. The pressure is returned as an array of time series at the sensor locations defined by `sensor.mask`. This is specified as a binary matrix (i.e., a matrix of 1's and 0's the same size as the computational grid) representing the grid points within the computational grid that will collect the data. The `sensor_data` is returned using MATLAB's standard column-wise linear matrix index ordering with the recorded data indexed as `sensor_data(sensor_position, time)`. The final pressure field over the complete computational grid can also be obtained by setting `sensor.record` to `{'p', 'p_final'}`. In this case, the output `sensor_data` is returned as a structure with the outputs appended as the structure fields `sensor_data.p` and `sensor_data.p_final`.

Compared to the first-order simulation functions `kspaceFirstOrder1D`, `kspaceFirstOrder2D`, and `kspaceFirstOrder3D`, `kspaceSecondOrder` is restricted to homogeneous media and has less functionality. However, it is exact for a homogenous absorbing medium, is more computationally efficient, and allows an initial pressure gradient to be specified.

## Inputs

|  |  |
| --- | --- |
| `kgrid` | k-Wave grid object returned by `kWaveGrid` containing Cartesian and k-space grid fields |
| `kgrid.t_array` | evenly spaced array of time values [s] (set to `'auto'` by `kWaveGrid`) |
|  |  |
| `medium.sound_speed` | homogeneous sound speed within the acoustic medium [m/s] |
| `medium.alpha_power` | power law absorption exponent |
| `medium.alpha_coeff` | power law absorption coefficient [dB/(MHz^y cm)] |
|  |  |
| `source.p0` | initial pressure within the acoustic medium |
| `source.dp0dt` | initial pressure gradient within the acoustic medium |
|  |  |
| `sensor.mask` | binary grid specifying where the pressure is recorded at each time-step |
| `sensor.record` | cell array of the acoustic parameters to record in the form `sensor.record = {'p'}`; valid inputs are:    `'p'` (acoustic pressure)    `'p_final'` (final pressure field at all grid points) |

## Optional Inputs

Optional 'string', value pairs that may be used to modify the default computational settings.

| Input | Valid Settings | Default | Description |
| --- | --- | --- | --- |
| `'ExpandGrid'` | *(Boolean scalar)* | `false` | Boolean controlling whether the grid size is expanded on two sides to delay the time before wave wrapping occurs. |
| `'MeshPlot'` | *(Boolean scalar)* | `false` | Boolean controlling whether `mesh` is used in place of `imagesc` to plot the pressure field. |
| `'PlotFrames'` | *(Boolean scalar)* | `false` | Boolean controlling whether the pressure field for each time step is plotted in a new window. |
| `'PlotFreq'` | *(integer numeric scalar)* | `10` | The number of iterations which must pass before the simulation plot is updated. |
| `'PlotScale'` | *(numeric two element vector)* | `[-1, 1]` | [min, max] values used to control the scaling for `imagesc` (visualisation). |
| `'PlotSim'` | *(Boolean scalar)* | `true` | Boolean controlling whether the simulation iterations are progressively plotted. |
| `'Smooth'` | *(Boolean scalar)* | `true` | Boolean controlling whether `source.p0` is smoothed using `smooth` before computation. |

## Outputs

If `sensor.record` is not defined by the user:

|  |  |
| --- | --- |
| `sensor_data` | time varying pressure recorded at the sensor positions given by `sensor.mask` |

If `sensor.record` is defined by the user:

|  |  |
| --- | --- |
| `sensor_data.p` | time varying pressure recorded at the sensor positions given by `sensor.mask` (returned if `'p'` is set) |
| `sensor_data.p_final` | final pressure field at all grid points within the domain (returned if `'p_final'` is set) |

## Examples

- Comparison Of Modelling Functions
- Setting An Initial Pressure Gradient
- Modelling Power Law Absorption

## See Also

`kspaceFirstOrder1D`, `kspaceFirstOrder2D`, `kspaceFirstOrder3D`, `kWaveGrid`, `smooth`
